# miR-27b antagonizes BMP signaling in early differentiation of human induced pluripotent stem cells

**DOI:** 10.1038/s41598-021-99403-9

**Published:** 2021-10-06

**Authors:** Jaeeun Lim, Eiko Sakai, Fuminori Sakurai, Hiroyuki Mizuguchi

**Affiliations:** 1 Laboratory of Biochemistry and Molecular Biology, Graduate School of Pharmaceutical Sciences, Osaka University, 1-6 Yamadaoka, Suita, Osaka 565-0871 Japan; 2 Laboratory of Hepatocyte Regulation, National Institute of Biomedical Innovation, Health and Nutrition, 7-6-8 Saito, Asagi, Ibaraki, Osaka 567-0085 Japan; 3 The Center for Advanced Medical Engineering and Informatics, Osaka University, 2-2 Yamadaoka, Suita, Osaka 565-0871 Japan; 4 Integrated Frontier Research for Medical Science Division, Institute for Open and Transdisciplinary Research Initiatives (OTRI), Osaka University, Osaka, Japan

**Keywords:** Molecular biology, Pluripotent stem cells, Stem-cell differentiation, Cell signalling

## Abstract

Human induced pluripotent stem (hiPS) cells are feasible materials for studying the biological mechanisms underlying human embryogenesis. In early embryogenesis, definitive endoderm and mesoderm are differentiated from their common precursor, mesendoderm. Bone morphogenetic protein (BMP) signaling is responsible for regulating mesendoderm and mesoderm formation. Micro RNAs (miRNAs), short non-coding RNAs, broadly regulate biological processes via post-transcriptional repression. The expression of miR-27b, which is enriched in somatic cells, has been reported to increase through definitive endoderm and hepatic differentiation, but little is known about how miR-27b acts during early differentiation. Here, we used miR-27b-inducible hiPS cells to investigate the roles of miR-27b in the undifferentiated and early-differentiated stages. In undifferentiated hiPS cells, miR-27b suppressed the expression of pluripotency markers [alkaline phosphatase (AP) and nanog homeobox (NANOG)] and cell proliferation. Once differentiation began, miR-27b expression repressed phosphorylated SMAD1/5, the mediators of the BMP signaling, throughout definitive endoderm differentiation. Consistent with the above findings, miR-27b overexpression downregulated BMP-induced mesendodermal marker genes [Brachyury, mix paired-like homeobox 1 (MIXL1) and eomesodermin (EOMES)], suggesting that miR-27b had an inhibitory effect on early differentiation. Collectively, our findings revealed a novel antagonistic role of miR-27b in the BMP signaling pathway in the early differentiation of hiPS cells.

## Introduction

Human embryonic stem (hES) and human induced pluripotent stem (hiPS) cells are widely used as alternative materials for studying human embryogenesis and its underlying signaling pathways, particularly its features recapitulating the developmental processes of human embryos in vitro. When epiblast cells start gastrulation and form mesoderm and endoderm, the activation of the nodal growth differentiation factor (NODAL), Wnt, bone morphogenetic protein (BMP), and other signaling pathways and transcription factors contribute to embryogenesis in a strictly controlled manner^1–3^. BMP is a signaling molecule of the transforming growth factor-beta (TGF-β) superfamily. BMP signaling has been shown to regulate both mesendoderm and mesoderm lineage differentiation in vitro and in vivo. In the in vitro differentiation of hES cells, BMP4 -treatment induced hES cells to generate mesendoderm and mesoderm^2,4^. Mouse embryos having the BMP type 2 mutant receptor were defective in mesoderm formation during early development^5,6^.

MicroRNAs (miRNAs) are short (18–25 nucleotide) non-coding RNAs that post-transcriptionally inhibit gene expression, generally by binding to the 3′ untranslated region (UTR) of mRNAs. They are differentially expressed depending on the developmental stage and cell type^7,8^. Expression profile of miRNAs have shown significant changes during fertilization and early development^9–11^, and an accumulation of evidence has revealed that miRNAs play pivotal roles in stabilizing the pluripotency and regulating the differentiation of hES/hiPS cells^12,13^. Since differentiation from hiPS cells recapitulates human embryogenesis, understanding how miRNA work in the differentiation of hiPS cells is important to reveal the mechanisms underlying the embryogenesis. miR-27b, a highly conserved miRNA across vertebrates, has been shown to regulate various target genes involved in signaling pathways^14,15^. In previous studies, miR-27b expression has been upregulated through differentiation from definitive endoderm to hepatocytes^8,16,17^ and the miR-23b cluster, which encodes miR-23b~27b~24, regulates hepatic differentiation by targeting SMAD3/4/5, the downstream molecules of the TGF-β superfamiliy^14^. In mouse oocyte, expression level of miR-27b was decreased after fertilization and remained low during subsequent cell proliferations^10^, suggesting that downregulation of miR-27b expression is a critical step to drive embryonic development. These observations suggested that miR-27b regulated differentiation processes by modulating signaling pathways. However, the precise roles of miR-27b in early differentiation are still elusive.

We previously generated hiPS cells expressing miR-27b in a doxycycline (dox) inducible manner (hiPS-AAVS1-27b) by using clustered regularly interspaced short palindromic repeats/CRISPR-associated 9 (CRISPR/Cas9), and thus revealed the negative effects of miR-27b on the maintenance of pluripotency. We also revealed that miR-27b inhibited the hepatic differentiation of hiPS cells^18^. In this report, we further examined how miR-27b affects the early differentiation of hiPS cells. Based on these results, we newly established the antagonistic role of miR-27b in BMP signaling.

## Results

### miR-27b suppressed the undifferentiated state of hiPS cells

Using the CRISP/Cas9 system, we previously generated a hiPS cell line that expresses miR-27b in a dox-inducible manner (hiPS-AAVS1-27b)^18^. We then used this inducible system to demonstrate that miR-27b negatively regulated the pluripotency of undifferentiated hiPS cells when they were cultured on mouse embryonic fibroblasts (MEFs). Recently, recombinant laminins, major components of basement membranes, were developed as alternative materials for the chemically defined culture condition of hES/hiPS cells to avoid contamination with undefined animal derivatives and to improve reproducibility. Laminin-511, a laminin isoform, has been reported to sustain the self-renewal and pluripotency of hES/iPS cells^19,20^. Therefore, in this study we first examined whether hiPS-AAVS1-27b cells cultured on recombinant laminin-511-coated plates showed the same phenotype as those cultured on MEFs.

**Figure 1:**
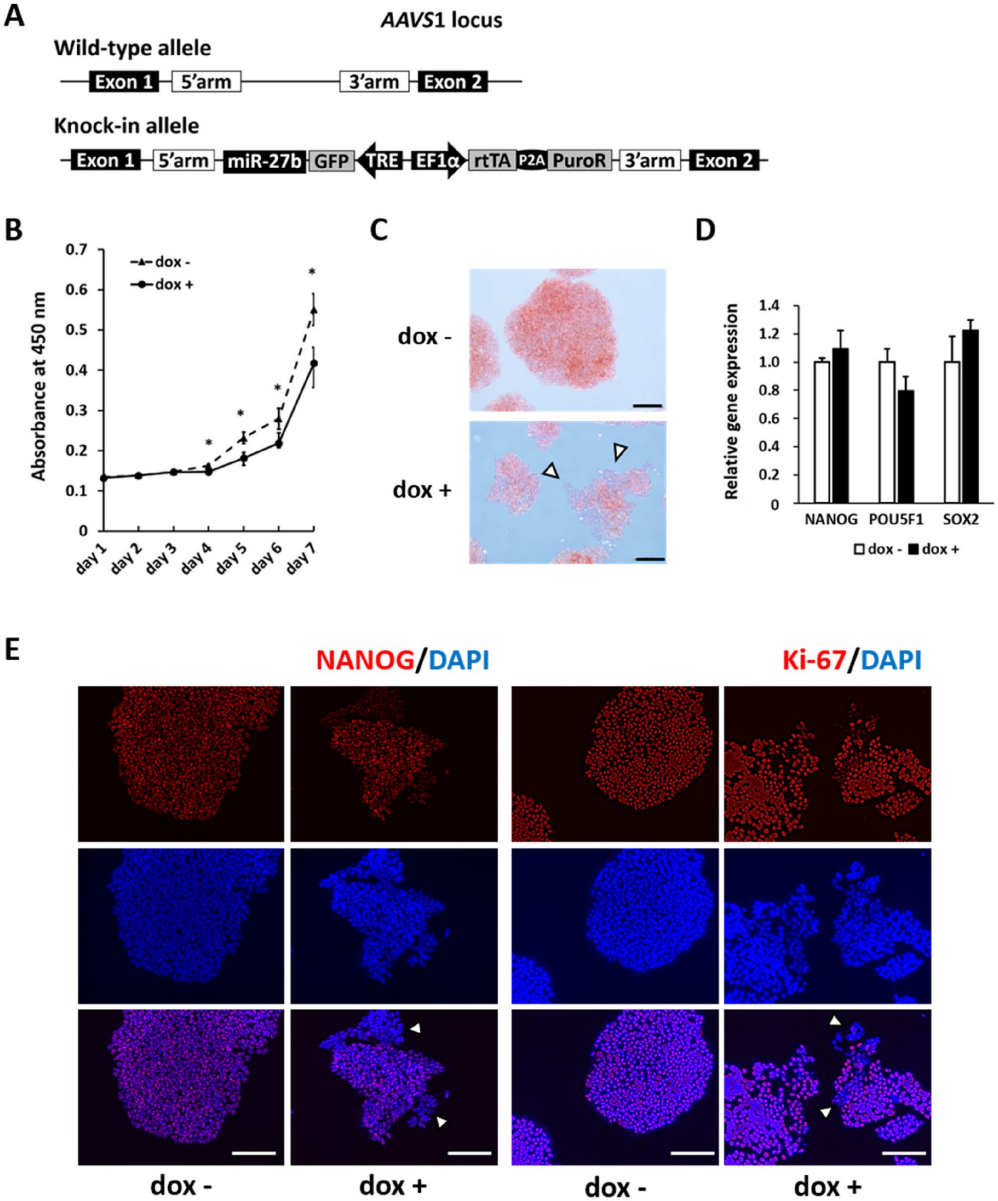
miR-27b suppressed pluripotency-related gene expression and proliferation of hiPS cells. (**A**) Schematic of AAVS1 alleles in hiPS-AAVS1-27b cells. Heterozygous integration of a dox-inducible miR-27b expression cassette into the AAVS1 locus was performed through homology directed repair (HDR). *AAVS1* adeno-associated virus integration site 1, *GFP* green fluorescent protein, *TRE* tetracycline response element, *rtTA* reverse tetracycline transactivator, *PuroR* puromycin N-acetyltransferase. (**B**) The proliferation rate of hiPS cells was analyzed by WST-8 assay. Doxycycline (dox) was added one day after seeding. Data are presented as mean ± SD (N = 3) (*p < 0.05) Student’s t test was performed. The representative result from two independent experiments is shown. (**C**) hiPS-AAVS1-27b cells were treated with dox (1 µg/ml) for 4 days and subjected to AP staining. Scale bars 200 µm. The representative result from two independent experiments is shown. (**D**) hiPS-AAVS1-27b cells were cultured with dox (1 µg/ml) for 5 days, and expression of pluripotency-related genes was analyzed by qRT-PCR (N = 3). The value of dox- was taken as 1.0. Data are presented as mean ± SD (N = 3). The representative data from three independent experiments is shown. (**E**) hiPS-AAVS1-27b cells were cultured with dox (1 µg/ml) for 5 days, and immunofluorescence analysis of Ki-67 and NANOG expression was carried out after culturing with dox for 5 days. Nuclei were counterstained with DAPI (blue). Scale bars 200 µm. The representative data from two independent experiments is shown.

The addition of dox successfully induced miR-27b expression in undifferentiated hiPS-AAVS1-27b cells cultured on laminin-511 (Fig. S1A). Undifferentiated hiPS-AAVS1-27b cells were cultured in the presence or absence of dox for 4 days, and then features of pluripotency were assessed. Undifferentiated hiPS cells generally form round colonies with well-defined edges, while the peripheral region of colonies exhibit epithelial cell-like morphologies once they start differentiation, indicating that peripheral region of colonies are more prone to differentiate^21–23^. Dox-treated undifferentiated hiPS-AAVS1-27b cells formed small colonies with skewed edges compared to the untreated control cells. In fact, these regions exhibited less staining of alkaline phosphatase (AP), a pluripotent stem cell marker, and the proliferation rates were significantly reduced compared to those of control cells cultured without dox (Fig. 1B,C). Moreover, while the overall expression levels of genes related to pluripotency [nanog homeobox (NANOG), POU class 5 homeobox 1 (POU5F1) and SRY-box transcription factor 2 (SOX2)] were unchanged in the qRT-PCR analysis (Fig. 1D), reduced protein expression levels of Ki-67, a proliferation marker, and of NANOG were observed at the edges of dox-treated undifferentiated hiPS colonies (Fig. 1E), suggesting that the undifferentiated state was lost at the edges. These results confirmed that the undifferentiated state of hiPS cells was suppressed by the induced expression of miR-27b even when cultured on laminin-511.

### miR-27b expression in early stages of differentiation reduced mesendodermal gene expression

**Figure 2:**
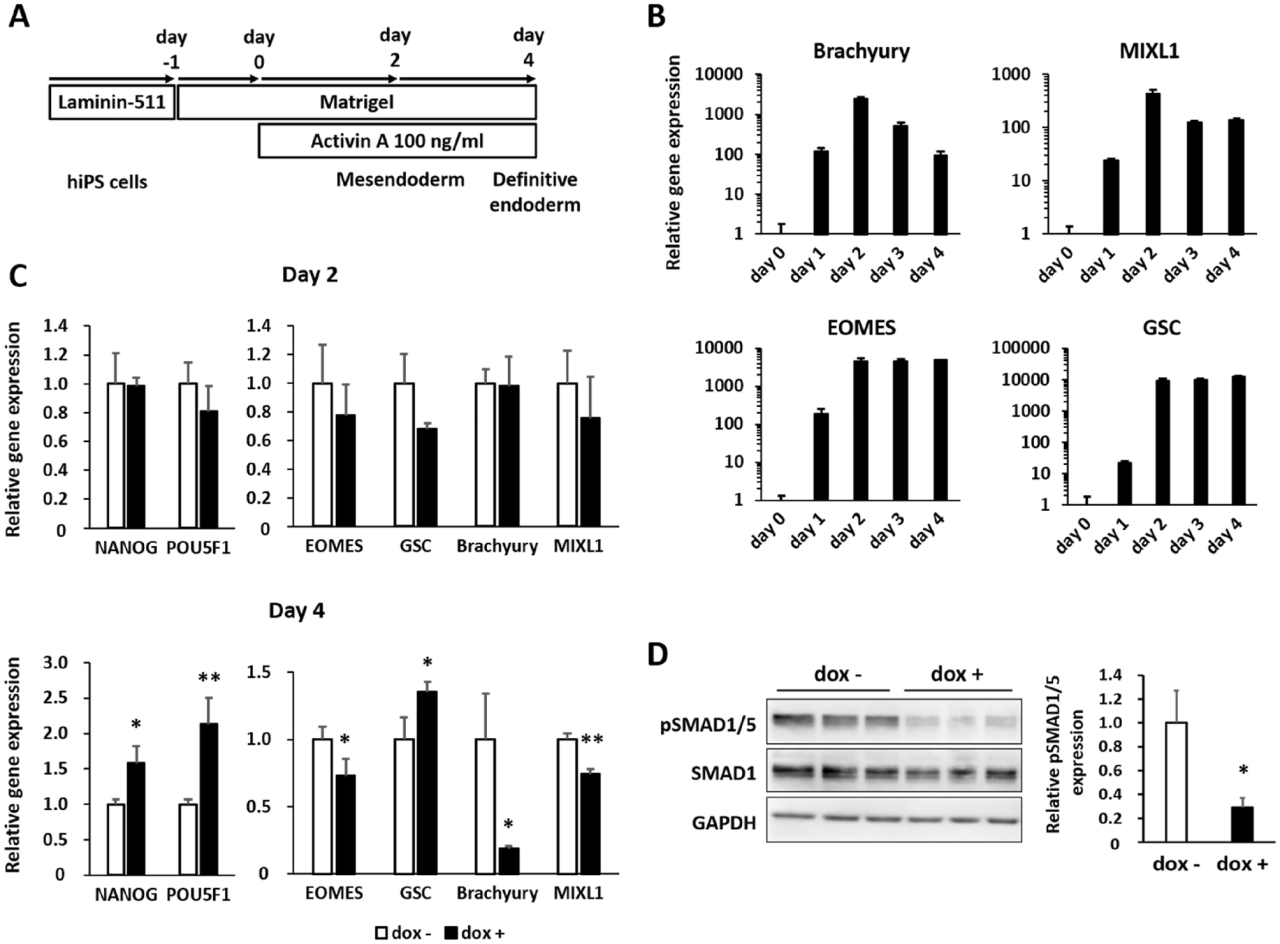
miR-27b expression induced in the differentiation stage reduced expression of mesendodermal genes. (**A**) Differentiation procedures. hiPS cells are seeded on Matrigel-coated plates and precultured for 1 day. Activin A (100 ng/ml) was added from day 0. hiPS cells were differentiated into mesendoderm at day 2 and definitive endoderm at day 4. (**B**) Expression levels of mesendodermal marker genes in hiPS-AAVS1-27b cells were analyzed by qRT-PCR in the time course of differentiation. The value of day 0 was taken as 1.0. Data are presented as mean ± SD (N = 3). The representative result from two independent experiments is shown. (**C**) Expression levels of differentiation marker genes in hiPS-AAVS1-27b cells were analyzed by qRT-PCR at day 2 (upper) and day 4 (lower) of differentiation. hiPS-AAVS1-27b cells were differentiated with or without dox (1 µg/ml). The value of dox- was taken as 1.0. Data are presented as mean ± SD (N = 3). Student’s t test was performed (*p < 0.05, **p < 0.01) The representative data from four independent experiments is shown. (**D**) Expression levels of phospho-SMAD1/5 protein in hiPS-AAVS1-27b cells at day 2 of differentiation were analyzed by Western blotting (left). Protein expression levels were quantitated and normalized by an internal reference, GAPDH (right). Original images are shown in Fig. S8A. The value of dox- was taken as 1.0. Data are presented as mean ± SD (N = 3). Student’s t test was performed (*p < 0.05). The representative result from four independent experiments is shown.

In general, the differentiation of hES/iPS cells proceeded by the activation of the appropriate signaling pathways, similar to the case in embryonic developmental processes^24,25^. During embryonic development, the primitive streak appears in epiblasts and migrates toward hyperblasts to generate both definitive endoderm and mesoderm^26,27^. Since previous studies have confirmed the feasibility of differentiation from the primitive streak toward endoderm and mesoderm in hES/iPS cells^28,29^, we analyzed the expression of marker genes that are prominently expressed in the primitive streak [eomesodermin (EOMES), Brachyury, mix paired-like homeobox 1 (MIXL1) and goosecoid homeobox (GSC)] in the directed hiPS differentiation toward definitive endoderm. hiPS-AAVS1-27b cells were subjected to differentiation toward the definitive endoderm according to the protocol shown in Fig. 2A. During 4 days of culture, the expression of these genes was dramatically increased at day 2. Brachyury and MIXL1 were particularly affected, reaching their highest levels around day 2 of differentiation (Fig. 2A,B). The subsequent drastic reductions in Brachyury and MIXL1, which have been reported to be the genes responsible for mesoderm formation^27,30^ from the primitive streak, indicated that definitive endoderm was formed around day 4. Based on this result, we considered cells on day 2 to be in the mesendodermal stage, where bi-potent cells with an ability to differentiate into both definitive endoderm and mesoderm were present. Therefore, we focused on days 2 (mesendoderm) and 4 (definitive endoderm) of differentiation.

We confirmed miR-27b expression in hiPS-AAVS1-27b cells throughout differentiation (Fig. S1B). The induced expression of miR-27b was 1.5–2-fold higher compared to the endogenous expression. Overexpression of miR-27b did not cause significant changes in Brachyury, MIXL1, or EOMES expression at day 2, but downregulated the expression of all three genes at day 4 (Fig. 2C). These expression patterns were similarly observed using the other hiPS clone (hiPS-AAVS1-27b-10) (Fig. S5B). The expression of GSC, however, was not suppressed, and was even slightly increased at day 4 in Fig. 2C, while it was suppressed in the other hiPS clone (Fig. S5A). Such an ambiguous expression of GSC seems to be related to the previous observation that its expression is kept through both mesendoderm and definitive endoderm stages^4,31,32^. On the other hand, the expression levels of marker genes related to pluripotency (NANOG and POU5F1) were not reduced, but rather were upregulated at day 4, suggesting that directed differentiation to mesendoderm was suppressed to some extent. Remarkably, miR-27b expression strongly inhibited expression of Brachyury, a specific marker for both mesendoderm and mesoderm progenitors^30^, suggesting that miR-27b selectively inhibited the decision toward mesodermal differentiation. Additional analysis for lineage-specific genes revealed the global decrease of mesodermal gene expression was induced by miR-27b expression, while endodermal gene expression was not much affected (Fig. S6). It seems that miR-27b expression blocked mesodermal differentiation from mesendoderm.

BMP drives the differentiation into mesoderm, whereas it reciprocally represses definitive endoderm differentiation in the primitive streak^6,28^. Therefore, we speculated that miR-27b repressed the activation of the BMP signaling pathway. When the phosphorylated SMAD1/5, transcriptional regulators of the BMP signaling pathway, were analyzed by Western blot analysis at day 2, miR-27b induction was found to have significantly repressed pSMAD1/5 (Fig. 2D). The suppression of pSMAD1/5 induced by miR-27b expression was also confirmed with hiPS-AAVS-27b-10 (Fig. S5B).

Taken together, these results indicate that miR-27b suppressed mesoderm differentiation from mesendoderm as well as the BMP signal.

### miR-27b antagonized the BMP signaling pathway during mesoderm/endoderm differentiation

**Figure 3:**
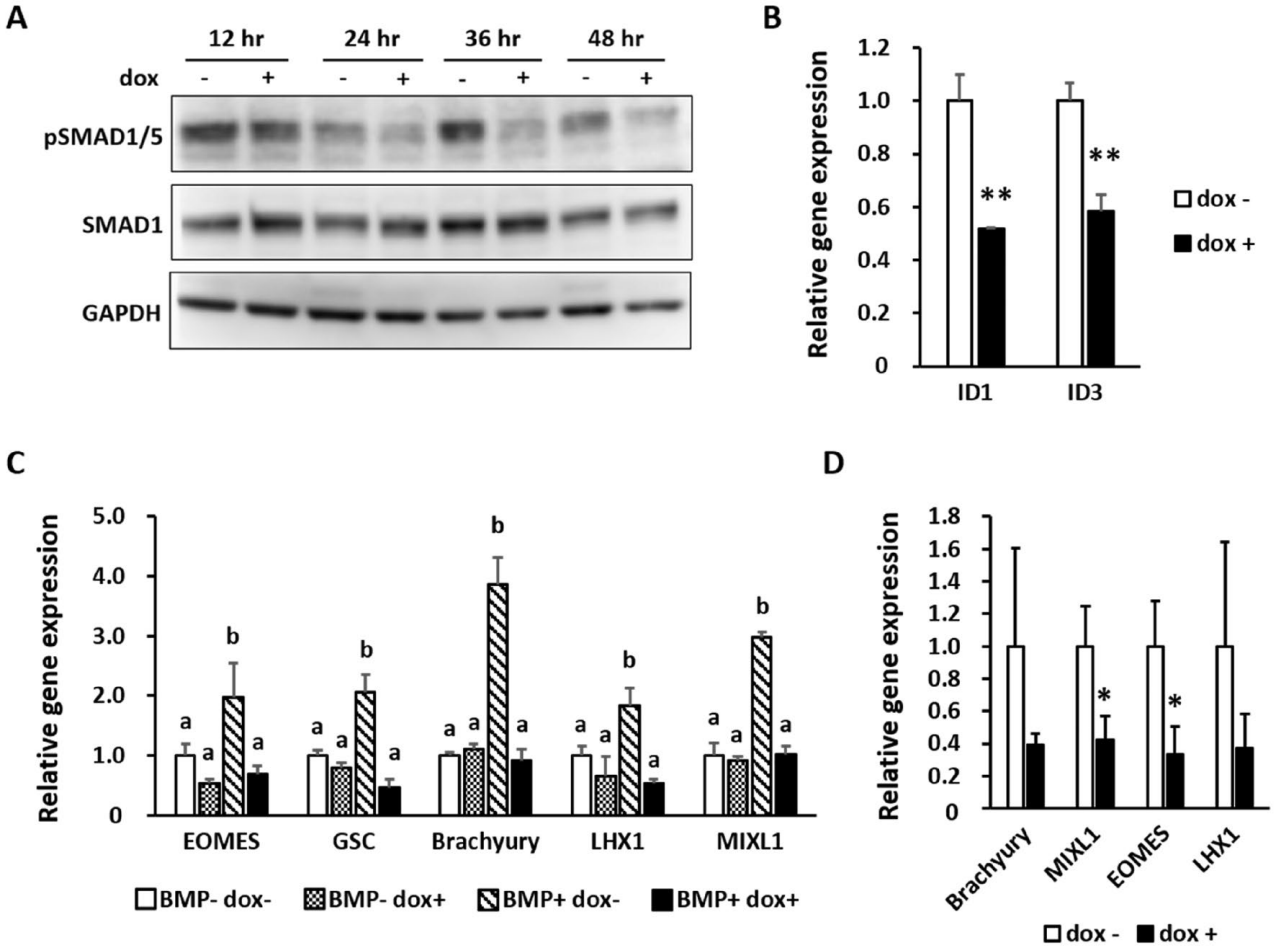
miR-27b antagonized the BMP signaling pathway during early differentiation. (**A**) hiPC-AAVS1-27b cells were differentiated into mesendoderm as in Fig. 2A with or without dox (1 µg/ml). Protein levels of phospho-SMAD1/5 were analyzed every 12 h of mesendoderm differentiation by Western blotting. Original images are shown in Fig. S8B. The representative result from two independent experiments is shown. (**B**) hiPC-AAVS1-27b cells were differentiated into mesendoderm as in Fig. 2A with or without dox (1 µg/ml). Expression levels of direct target genes of BMP signaling were analyzed by qRT-PCR. The value of dox- was taken as 1.0. Data are presented as mean ± SD (N = 3). Student’s t test was performed (**p < 0.01). The representative data from three independent experiments is shown. (**C**) hiPC-AAVS1-27b cells were differentiated into mesendoderm as in Fig. 2A with or without dox (1 µg/ml) and BMP4 (10 ng/ml), and mesendodermal marker genes were analyzed by qRT-PCR. The value of BMP-dox- was taken as 1.0. Data are presented as mean ± SD (N = 3). One-way ANOVA was performed, followed by Tukey’s post-hoc test. Groups labeled ‘b’ are significantly different from labeled ‘a’ (p < 0.05). The representative data from two independent experiments is shown. (**D**) Gene expression levels in EBs were analyzed 3 days after seeding by qRT-PCR. Dox (1 µg/ml) was added starting from 24 h after seeding. The value of dox- was taken as 1.0. Data are presented as mean ± SD (N = 3). Student’s t test was performed (*p < 0.05) The representative data from four independent experiments is shown.

Our results revealed that phosphorylation of SMAD1/5 was repressed in hiPS cells that were induced to express miR-27b. Since the striking temporal activation of BMP signaling commits cells to differentiate into the anterior primitive streak^28^, we further examined the phosphorylation levels of SMAD1/5 from 12 to 48 h of differentiation. Phosphorylation levels of SMAD1/5 increased transiently and remarkably at 36 h of differentiation without dox, while miR-27b abrogated this spike and repressed phosphorylation persistently until 48 h (Fig. 3A). This result suggested that miR-27b disturbed differentiation by blocking the temporal spike and the subsequent sustained activation of BMP signaling. Consistently, induction of miR-27b expression in mesendoderm also reduced the expression of direct target genes of the BMP signaling pathway (inhibitor of DNA binding (ID) 1, ID3) (Fig. 3B).

To confirm the antagonizing effect of miR-27b on BMP signaling, we exogenously added BMP4 into the culture medium from day 0 to day 2 and analyzed gene expression by qRT-PCR. The addition of BMP4 significantly enhanced the expression of mesendodermal marker genes (Brachyury, MIXL1, EOMES, etc.), while miR-27b expression counteracted these effects (Fig. 3C). The downregulations of mesendodermal marker genes and of pSMAD1/5 were not observed in wild-type hiPS cells cultured with dox (Fig. S2A,B), validating that miR-27b, but not dox treatment, induced the antagonizing effects on BMP signaling. In addition, we examined the effects of miR-27b in another in vitro differentiation system by subjecting hiPS-AAVS1-27b cells to embryoid body (EB) formation. We observed a similar downregulation of mesendodermal genes when miR-27b was induced in EBs (Fig. 3D), suggesting that the inhibitory effects of miR-27b on BMP signals were not specific for the directed differentiation.

### miR-27b directly regulated genes related to the BMP signaling pathway

**Figure 4:**
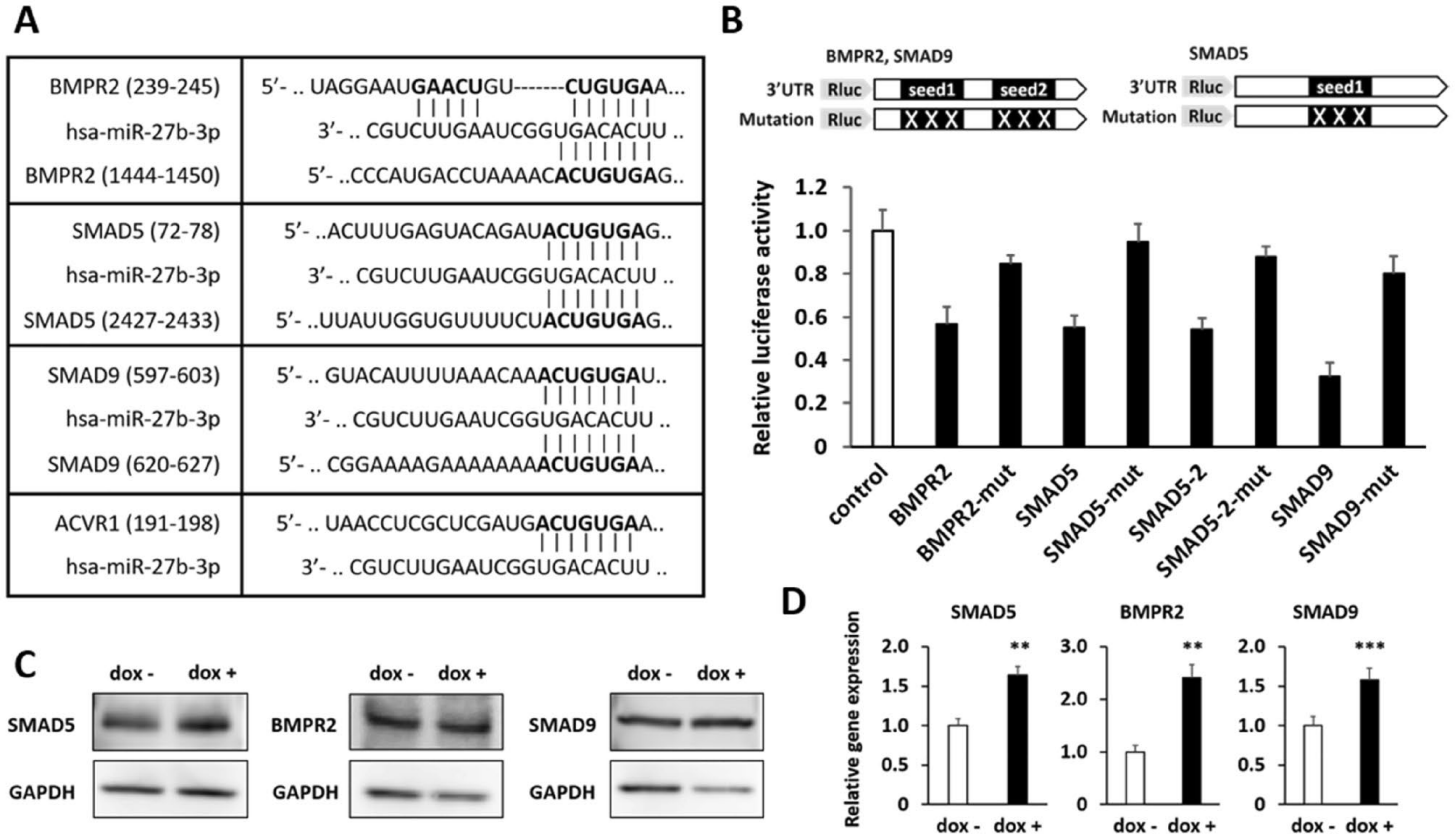
miR-27b indirectly regulated the BMP signaling pathway. (**A**) Predicted pairings of the 3′UTRs of target genes to miR-27b-3p are aligned. (**B**) Luciferase reporter plasmids were co-transfected with pHM-U6-pre-miR-27b into HEK293 cells, and luciferase activity was measured 48 h after transfection. The data from cells co-transfected with the control reporter vector without any 3′UTR fragment downstream of Renilla luciferase gene are taken as 1.0. Data are presented as mean ± SD (N = 3). The student’s t test was performed (***p < 0.001). Experiments were performed twice in triplicate. The representative result from two independent experiments is shown. (**C**,**D**) hiPS-AAVS1-27b cells were differentiated into mesensoderm as in Fig. 2A with or without dox (1 µg/ml). Protein (**C**) and mRNA (**D**) expression levels of BMPR2, SMAD5 and SMAD9 in the cells at day 2 (mesendoderm) were analyzed by Western blotting and qRT-PCR, respectively. Original images of Western blotting are shown in Fig. S8C. The value of dox- are taken as 1.0. Data are presented as mean ± SD (N = 3). The student’s t test was performed (*p < 0.05). qRT-PCR were performed in triplicate and repeated at least twice. The representative images of western blotting from three independent experiments is shown.

We demonstrated that miR-27b repressed BMP signaling in the early differentiation of hiPS cells. To test whether miR-27b directly regulates BMP signaling, we identified putative target genes, which were targets of miR-27b as well as genes involved in BMP signaling, using the combination of TargetScanHuman7.2, a target prediction tool, and DAVID, a functional annotation tool, yielding SMAD5, SMAD9, activin A receptor type 1 (ACVR1), and bone morphogenetic protein receptor type 2 (BMPR2) as candidates. ACVR1 and BMPR2, BMP receptor type 1 and type 2, respectively, are transmembrane serine/threonine kinase receptors and bind to BMPs. SMAD5 and SMAD9 are transcription factors, which are downstream mediators of the BMP receptors. Once phosphorylated by activation of BMP receptors, SMAD5/9 translocate into nuclei and function as transcription factors. To examine whether they are the direct targets of miR-27b, the 3′UTR regions of these genes were cloned downstream of the Renilla luciferase gene in the psiCHECK-2 vector and co-transfected with an miR-27b-expression vector (pHM5-U6-pre-miR-27b^33^) into HEK293 cells, then dual luciferase assays were carried out 48 h after transfection. Renilla luciferase expression levels in reporter vectors containing the 3′UTRs of SMAD5, SMAD9, and BMPR2 were downregulated to around 50% compared to the control reporter vector (Fig. 4A,B), while the reporter plasmid containing the 3′UTR of ACVR1 was not (Fig. S3). The reporter activity was restored by introducing mutations within the seed-matched regions, indicating that SMAD5, SMAD9, and BMPR2 were direct target genes of miR-27b (Fig. 4B). Next, the mRNA and protein expression levels of these genes were analyzed at day 2 of differentiation from hiPS-AAVS1-27b cells by qRT-PCR and Western blotting, respectively. Unexpectedly, mRNA and protein expressions in hiPS-AAVS1-27b cells cultured with dox were not suppressed (Fig. 4C,D). These showed that miR-27b did not regulate them during differentiation. The transcription of SMAD5, SMAD9, and BMPR2 were even promoted compared to the control cells cultured without dox at day 2, suggesting the occurrence of feedback activation caused by inhibition of the BMP signaling (Fig. 4D).

These results suggested that miR-27b suppressed BMP signaling in both a direct and an indirect manner.

## Discussion

In this study, we investigated the role of miR-27b in undifferentiated and mesoderm/endoderm-differentiated hiPS cells by using a dox-dependently miR-27b-inducible hiPS cell line. miR-27 is known to have several target genes involved in regulating members of the TGFβ superfamily^14,17^, which play pivotal roles in both pluripotency and differentiation. In fact, miR-27b inhibits the expression of pluripotency-associated genes in embryonal carcinoma (EC) cells^17^. Consistent with these finding, we previously reported that miR-27b strongly inhibited the pluripotency of hiPS cells cultured on MEFs^18^.

Laminin-511, a laminin isoform, is known to support the proliferation and self-renewal of hES/ hiPS cells^19,20,34^ and is considered a promising extracellular matrix for the xeno-free culture of hES/hiPS cells. Therefore, we tested whether miR-27b still inhibits the pluripotency of hiPS cells cultured on laminin-511. Induced miR-27b expression caused size- and morphological changes in hiPS colonies, in addition to decreasing the proliferation rate. Consistent with this finding, the expressions of pluripotency-related proteins, such as NANOG and AP, were reduced at the edges of hiPS cell colonies (Fig. 1). However, under the feeder-free condition using laminin-511 as a matrix, we did not observe the strong inhibition of pluripotency related genes (NANOG and POU5F1) or drastic morphological changes in colonies with ambiguous edges, which we did observe in hiPS cells cultured on MEFs in our previous study^18^. Laminin-511 is composed of α5, β1, and γ1 chains, and the adhesion of hES/hiPS cells depends on α6β1 integrin, which is abundantly expressed in hES/hiPS cells and supports their self-renewal^19,34^. Therefore, undifferentiated hiPS cells cultured on laminin-511 might be supported by integrin signaling and resistant to differentiation^20^; and culturing on laminin-511 might compete with and overcome the effects of miR-27b expression. The detailed mechanisms underlying these actions should be elucidated.

During differentiation, miR-27b repressed the expression levels of pSMAD1/5, which are downstream molecules of BMP signaling, and this pSMAD1/5 repression was accompanied by the suppression of mesendodermal/mesodermal genes. Moreover, miR-27b reversed the upregulation of the expression of mesendodermal marker genes (Brachyury, MIXL1, etc.), which the addition of BMP4 robustly induced, suggesting that miR-27b antagonized BMP signaling and therefore suppressed BMP-mediated mesendoderm formation. Since BMP signaling plays reciprocal roles in endoderm and mesoderm differentiation from the primitive streak^28^, miR-27b expression might commit cells to endoderm differentiation from mesendoderm by suppressing BMP signaling (Figs. S6, S7).

Focusing on BMP signaling, we used TargetScanHuman7.2 and DAVID to screen for putative miR-27b target genes and successfully identified SMAD5, SMAD9, and BMPR2 as possible direct target genes, which were verified by luciferase reporter assays (Fig. 4). However, the mRNA and protein expression levels of these genes were not reduced in mesendoderm at day 2 of differentiation, suggesting that miR-27b indirectly regulated BMP signaling during differentiation. Since differentiation from hiPS cells is orchestrated by various signaling pathways such as those of TGFβ and NODAL, those pathways influence each other during differentiation^2,35^. The BMP signal could be modulated through the regulation of other signals, as we observed an increase in pSMAD2 by miR-27b expression (Fig. S4). Alternatively, the differentiating cells may comprise a heterologous population in which only a small portion of cells are differentiated toward the mesodermal lineages via changes in the phosphorylated form of SMAD1/5. Therefore, suppression of target genes caused by miR-27b expression in such cells might be buried beneath the expression of those target genes in the vast majority of cells.

In conclusion, our data revealed that miR-27b antagonized BMP signaling at early differentiation stages and arrested mesodermal differentiation. We identified three target genes of miR-27b involved in the BMP signaling cascade, although we could not show that they were repressed in this differentiation period. These findings should help to further elucidate the biological pathways underlying human embryogenesis. In addition, in the light of the increasing requirement of hiPS cells for regenerative medicine and tissue engineering, our findings that manipulation of miR-27b expression could modify the hiPS cell differentiation would contribute to such fields. Manipulation of miR-27b in early differentiation might be also important in clinical fields. The previous observations that expression level of miR-27b is significantly downregulated during the first division of mouse zygote^10^, combined with our observations that upregulated expression of miR-27b compromises the early differentiation of hiPS cells, strongly suggested that strictly downregulated miR-27b expression is a key event for early embryonic development. Interestingly, secreted miR-27b in the culture media of human blastocyst with successful pregnancy was reduced compared to that of blastocysts with failed pregnancy^36^, suggesting potential relevance of miR-27b as a therapeutic target of human infertility. It remains elusive whether the inhibitory effects of miR-27b on early differentiation stages are involved. Additional studies would be required to reveal the molecular mechanisms by which miR-27b regulates hiPS cell differentiation via BMP signaling and to utilize miR-27b in manipulation of early developmental processes and in clinical applications.

## Materials and methods

### Human iPS cell culture and differentiation

A hiPS-AAVS1-27b cell line containing heterozygously knocked-in dox-inducible miR-27b expression cassette within the AAVS1 locus was previously generated^18^. hiPS-AAVS1-27b and human iPS cell lines (Tic, human iPS cell line obtained from JCRB Cell Bank, Japan) were cultured on the laminin-511 E8 fragment (iMatrix-511 silk, Nippi, Tokyo, Japan) with StemFit AK02N (ReproCELL, Kanagawa, Japan). For in vitro definitive endoderm differentiation, hiPS cells were dissociated into single cells by treatment with TrypLE Select Enzyme (Thermo Fisher Scientific, Waltham, MA) and seeded onto plates coated with BD Matrigel Matrix Basement Membrane Growth Factor Reduced (Corning, Corning, NY) at a density of 3.0 × 10^5^ cells/well (24 well plate) or 6.0 × 10^5^ cells/well (12 well plate). Cells were precultured with StemFit AK02N, supplemented with 10 μM Rock inhibitor (Y-27632, Sigma-Aldrich, St. Louis, MO) for 1 day. The definitive endoderm cells were induced by culturing in RPMI 1640 medium (Sigma-Aldrich) supplemented with 1xB27 Supplement Minus Vitamin A (Thermo Fisher Scientific) and 4 mM GlutaMAX (Thermo Fisher Scientific) in the presence of 100 ng/ml Activin A (R&D Systems, Minneapolis, MN) for 4 days. Doxycycline (1 μg/ml) (TAKARA BIO, Shiga, Japan) or BMP4 (10 ng/ml) (R&D Systems) was added to RPMI-B27 medium.

### Embryoid body (EB) formation

To generate EBs, hiPS cells were dissociated into single cells by treatment with TrypLE Select Enzyme (Thermo Fisher Scientific), seeded in a low-attachment 96 U plate (Thermo Fisher Scientific) at 5 × 10^3^ cells/well, and cultured in EB medium (D-MEM/F-12, GlutaMAX supplement (Thermo Fisher Scientific) containing 20% fetal bovine serum, 55 μM β-mercaptoethanol (Thermo Fisher Scientific), 0.1 mM MEM-Non Essential Amino Acids (Thermo Fisher Scientific). Medium was changed every other day.

### RNA isolation and quantitative RT-PCR (qRT-PCR)

Total RNA was isolated from hiPS cells cultured on plates and EBs using Sepasol-RNA I Super G (NACALAI TESQUE, Kyoto, Japan) and ISOGEN-2 (NIPPON GENE, Tokyo, Japan), respectively, according to the manufacturer’s instructions. RNA was used to synthesize cDNAs with a Superscript VILO cDNA synthesis kit (Thermo Fisher Scientific). qRT-PCR was carried out with StepOnePlus real-time PCR system (Thermo Fisher Scientific) using Fast SYPR Green Master Mix (Thermo Fisher Scientific). Results were analyzed with ∆∆Ct method, normalized by the internal reference, glyceraldehyde-3-phosphate dehydrogenase (GAPDH). The primer sequences are described in Table S1. Heatmap of expression profile was generated using Heatmapper (http://www.heatmapper.ca/).

### miRNA TaqMan assay

Taqman MicroRNA Assay Kits (assay ID 000409, Thermo Fisher Scientific) was used for quantification of miR-27b expression according to the manufacturer’s instructions. Briefly, 5 ng of total RNA was used to perform reverse-transcription (RT) and 1 μl of RT products out of 7.5 μl of total reaction mixture was used for qRT-PCR. Results were analyzed with ∆∆Ct method normalized by the internal control, RNU48 (assay ID 001006, Thermo Fisher Scientific).

### WST-8 assay

hiPS cells were seeded in 24 well plate at 1.5 × 10^3^ cells/well and dox (1 µg/ml) was added on the next day. Cell viability was measured every day with Cell Counting Kit-8 (DOJINDO LABORATORIES, Kumamoto, Japan) according to the manufacturer’s instructions.

### Alkaline phosphatase (AP) staining

hiPS cells were cultured for 4 days with dox (1 µg/ml) and AP staining was performed by Red-Color AP staining Kit (System Biosciences, Palo Alto, CA) according to the manufacturer’s instructions.

### Dual luciferase assay

Putative target genes and binding regions of hsa-miR-27b were identified using TargetScanHuman7.2 (http://www.targetscan.org/vert_71/) and Functional Annotation tool DAVID (https://david.ncifcrf.gov/summary.jsp) with GOTERM_BP_DIRECT list. The 3′UTR fragments of ACVR1, BMPR2, SMAD5, and SMAD9 were amplified from the genomic DNA of hiPS cells using PrimeSTAR Max DNA Polymerase (TAKARA BIO) and the primers listed in Table S2, and cloned into the downstream region of the Renilla luciferase gene in psiCHECK-2 vector (Promega, Madison, WI) digested with XhoI/NotI. Site-specific mutation was introduced by PrimeSTAR Mutagenesis Basal Kit (TAKARA BIO) and the primers in Table S2 according to the manufacturer’s instructions. The plasmid for miR-27b expression was generated before (pHM5-U6-pre-miR-27b)^33^.

Each reporter plasmid was co-transfected with pHM5-U6-pre-miR-27b into HEK293 cells and luciferase activities were measured 48 h after transfection using Dual-Luciferase Reporter Assay system (Promega) according to the manufacturer’s instructions. The Renilla luciferase activity was normalized to the Firefly luciferase activity. The Renilla/Firefly luciferase activity of cells co-transfected with psiCHECK-2 and pHM5-U6-pre-miR-27b was taken as 1.

### Immunofluorescence staining

The cells were fixed with 4% paraformaldehyde (PFA, FUJIFILM Wako Pure Chemical Corporation, Osaka, Japan) in PBS for 30 min at room temperature. After permeabilized with PBS containing 0.2% Triton X-100 (Sigma-Aldrich) and 2% BSA for 45 min at 4 °C, the cells were incubated with a primary antibody overnight at 4 °C, and subsequently incubated with a secondary antibody for 1 h at room temperature. The nuclei were stained with DAPI for 5 min at room temperature. All the antibodies and the dilution are listed in Table S3.

### Western blot analysis

The cells were lysed with RIPA buffer (Thermo Fisher Scientific) containing a protease inhibitor cocktail (Sigma-Aldrich) and a phosphatase inhibitor cocktail (NACALAI TESQUE). The lysates were analyzed by SDS-PAGE, followed by electronic transfer onto a polyvinylidene fluoride (PVDF) membrane. After blocking with 5% skim milk or 5% BSA in Tris-buffered saline (TBS) containing 0.1% Tween 20 for 1 h at room temperature, the membranes were incubated with primary antibodies overnight at 4 °C, followed by incubation with secondary antibodies for 1 h at room temperature. The signals were visualized by Chemi-Lumi One Super (NACALAI TESQUE) and LAS-4000 imaging system (FUJIFILM, Tokyo, Japan). The intensity of each signal was quantified by imageJ. All the antibodies and the dilution are listed in Table S4.

### Statistical analysis

Statistical analysis was performed with two-tailed Student’s t test for paired comparisons or One-way ANOVA Tukey’s post-hoc test for multiple comparisons.

## Supplementary Information

Supplementary Information.
