# Supplementary material for: miR-27b antagonizes BMP signaling in early differentiation of human induced pluripotent stem cells
Source: Sci Rep. 2021 Oct 6;11:19820. doi: 10.1038/s41598-021-99403-9 (PMC8494899; doi:10.1038/s41598-021-99403-9)
Supplement: Supplementary file 1 — Supplementary Information. [file 41598_2021_99403_MOESM1_ESM.pdf]

Supplementary Information

Title

miR-27b antagonizes BMP signaling in early differentiation of human induced pluripotent stem cell.

Authors

Jaeeun Lim, Eiko Sakai, Fuminori Sakurai and Hiroyuki Mizuguchi

Supplementary Table

Supplementary Table S1

Supplementary Table S2

Supplementary Table S3

Supplementary Table S4

Supplementary Figure

Supplementary Figure S1

Supplementary Figure S2

Supplementary Figure S3

Supplementary Figure S4

Supplementary Figure S5

Supplementary Figure S6

Supplementary Figure S7

Supplementary Figure S8

Supplementary Table S1. Primer sets for qRT-PCR

gene	Forward primer (5' - 3')	Reverse primer (5' - 3')
GAPDH	GGTGGTCTCCTCTGACTTCAACA	GTGGTCGTTGAGGGCAATG
NANOG	AGAAGGCCTCAGCACCTAC	GGCCTGATTGTTCCAGGATT
SOX2	GGCAGCTACAGCATGATGATGCAGGAGC	CTGGTCATGGAGTTGTACTGCAGG
POU5F1	CTTGAATCCCGAATGGAAAGGG	GTGTATATCCCAGGGTGATCCTC
GSC	TCTCAACCAGCTGCACTGTC	CGTTCTCCGACTCCTCTGAT
MIXL1	GGCGTCAGAGTGGGAAATCC	GGCAGGCAGTTCACATCTAC
Brachyury	TATGAGCCTCGAATCCACATAGT	CCTCGTTCTGATAAGCAGTCAC
ID1	CTCTACGACATGAACGGCTGT	TGCTCACCTTGCGGTTCTG
ID3	TCAGCTTAGCCAGGTGGAAATC	TGGCTCGGCCAGGACTAC
LHX1	CCTGGACCGCTTTCTCTTGAA	ACCGAAACACCGGAAGAAGTC
EOMES	CAGCACCACCTCTACGAACA	CGCCACCAAACTGAGATGAT
FOXA2	GCGACCCCAAGACCTACAG	GGTTCTGCCGGTAGAAGGG
SOX17	GTGGACCGCACGGAATTTG	GGAGATTCACACCGGAGTCA
HHEX	CACCCGACGCCCTTTTACAT	GAAGGCTGGATGGATCGGC
CER1	GGATGGCCGCCAGAATCAG	TGGCACTGCGACAAACAGAT
GATA4	CATCAAGACGGAGCCTGGCC	TGACTGTCGGCCAAGACCAG
GATA6	CCATGACTCCAACTTCCACC	ACGGAGGACGTGACTTCGGC
FOXH1	CTTCCCTGAAGAAGGGGAAC	GGAAGAAGAGGTACCTGCGA
CXCR4	ACGCCACCAACAGTCAGAG	AGTCGGGAATAGTCAGCAGGA
KDR	GGCCCAATAATCAGAGTGGCA	TGTCATTTCCGATCACTTTTGGA
PDGFR	TTGAAGGCAGGCACATTTACA	GCGACAAGGTATAATGGCAGAAT
MESP2	CACGACCACTGGATCTTCGC	AACCCGACGAATCGGAGGA
SNAI1	TCGGAAGCCTAACTACAGCGA	AGATGAGCATTGGCAGCGAG
SNAI2	CGAACTGGACACACATACAGTG	CTGAGGATCTCTGGTTGTGGT
HLX	ATCTCACTTCCCTGCTAACCG	AGAAGCCTCGTTAATGGGATCT
MEOX1	CTTCACACGCTTCCACTTCA	GCACTGCCAATGAGACAGAG
FOXF1	AGTCCCCAATGCAAAGACAC	TCAGCAGAATTCCTGTGTGG

Supplementary Table S2. Primer sets for plasmid constructions

gene	region of 3'UTR	Forward primer (5' - 3')	Reverse primer (5' - 3')
ACVR1	7-835	CATAGTGTCAAGAAGGAAG	GTTACAGTCTACACACATAC
BMPR2	55-1538	CGACCTCGAGAACTCCCTATTCTCTTAAGCG	GGTGGCGGCCGCAGTTTAGTCTCAGTTTGCTC
SMAD5	-145-543	CGACCTCGAGGGGTTGGGGAGCAGAATATC	GGTGGCGGCCGCTACAGCAGTGCATAGTGTTC
SMAD5-2	1951-3089	CGACCTCGAGGTTTGACTTTCCACTTTGTCC	GGTGGCGGCCGCCAGGCAATACTGACCAGACC
SMAD9	249-980	CGACCTCGAGGAGAGGCGATATTGTCAACAG	GGTGGCGGCCGCGTGCCCTTCCCAGGAGACTG
BMPR2 mut1		CTGTC**A**G**C**G**T**AATGTTTTCAAGCCTATG	ACATT**A**C**G**C**T**GACAGTTCATTCCTATATC
BMPR2 mut2		AAACA**G**T**A**T**C**AGGAAAAATCATTCAAGTG	TTCCT**G**A**T**A**C**TGTTTTAGGTCATGGGAAG
SMAD5 mut		AGATA**G**T**A**T**C**AGCTTACATTGAAAACAGA	AAGCT**G**A**T**A**C**TATCTGTACTCAAAGTTTC
SMAD5-2 mut		TTCTA**G**T**A**T**C**AGTTAATTAAAAATTGTTT	TAACT**G**A**T**A**C**TAGAAAACACCAATAAAAC
SMAD9 mut1		ACAAAC**G**G**C**GATCGGAAAAGAAAAAAAAC	CCGATC**G**C**C**GCTTGTTTAAAATGTACCAC
SMAD9 mut2		AAAA**G**C**G**G**C**GAAGCCAAAAGTCATGTTC	GGCTTC**G**C**C**G**C**TTTTTTTCTTTTCCGATC

underline; seed region, **bold**; mutation

Supplementary Table S3. Antibodies for immunofluorescence staining

antigen	type	Dilution	company
NANOG	mouse	1:100	Santa Cruz Biotechnology
Ki-67	rabbit	1:500	Abcam
Alexa Fluor 594 anti-mouse IgG	donkey	1:500	Thermo Fisher Scientific
DAPI		1:1000	NACALAI TESQUE

Supplementary Table S4. Antibodies for Western blotting

antigen	type	Dilution	blocking buffer	company
GAPDH	rabbit	1:3000	5% BSA/TBST	Trevigen
phospho-SMAD1/5	rabbit	1:1000	5% BSA/TBST	Cell Signaling Technologies
SMAD1	rabbit	1:1000	5% BSA/TBST	Cell Signaling Technologies
SMAD5	rabbit	1:1000	5% Skim milk/TBST	Proteintech
phospho-SMAD2	rabbit	1:1000	5% BSA/TBST	Cell Signaling Technologies
SMAD2/3	rabbit	1:1000	5% BSA/TBST	Cell Signaling Technologies
SMAD9	rabbit	1:1000	5% Skim milk/TBST	Proteintech
BMPR2	rabbit	1:1000	5% Skim milk/TBST	Proteintech
Anti-rabbit IgG, HRP-linked Antibody	goat	same as 1st antibody	same as 1st antibody	Cell Signaling Technologies

Supplementary Figure S1

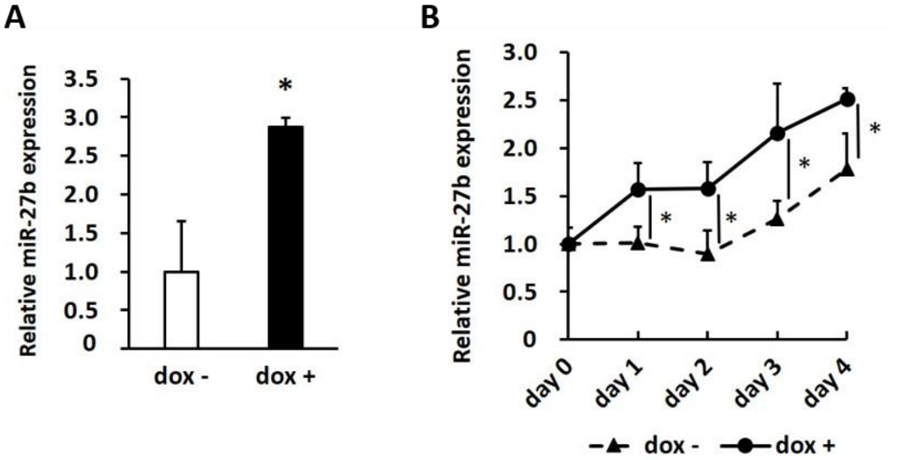

Figure S1. miR-27b expression in dox-treated undifferentiated hiPS-AAVS1-27b cells.

(A) miR-27b expression levels in undifferentiated hiPS-AAVS1-27b cells were analyzed by qRT-PCR after 48 hr culturing with or without dox. The value of dox- are taken as 1.0. Data are presented as mean±SD (N=3). The student's t-test was performed (**$p<0.01$). The representative result from two independent experiments is shown.

(B) miR-27b expression levels during definitive endoderm differentiation were analyzed by qRT-PCR. The value of day 0 are taken as 1.0. Data are presented as mean±SD (N=3). The student's t-test was performed (*$p<0.05$). The representative result from two independent experiments is shown.

Supplementary Figure S2

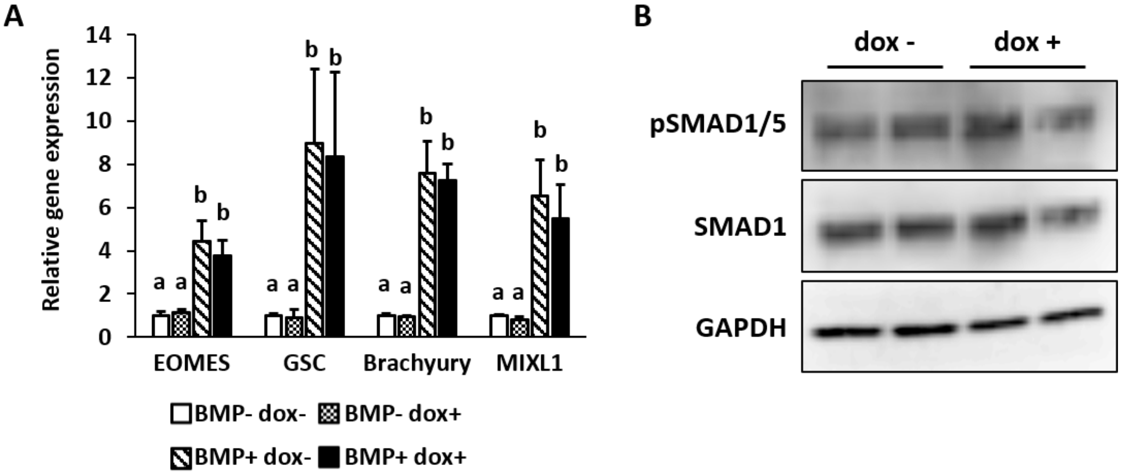

Figure S2. Dox treatment did not repress BMP signals in wild-type human iPS cells.

(A) Wild-type hiPS cells (Tic) were differentiated into mesendoderm as in Fig. 2A with or without dox (1 μg/ml) and BMP4 (10 ng/ml), and mesendodermal marker genes were analyzed by qRT-PCR. The value of BMP-dox- are taken as 1.0. Data are presented as mean±SD (N=3). One-way ANOVA was performed, followed by Tukey's post-hoc test. Groups labeled 'b' are significantly different from labeled 'a' ($p < 0.01$). The experiment was performed in triplicate.

(B) Wild-type hiPS cells (Tic) were differentiated into mesendoderm as in Fig. 2A with or without dox (1 μg/ml), and phospho-SMAD1/5 was detected by Western blotting. Original images are shown in Fig. S8D. Representative image from triplicate experiment.

Supplementary Figure S3

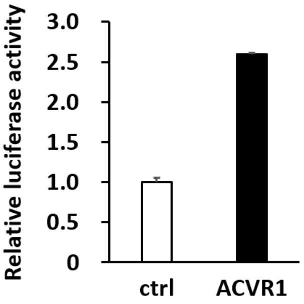

Figure S3. miR-27b did not target the 3'UTR of ACVR1. Luciferase reporter plasmid containing 3'UTR of ACVR1 was co-transfected with pHM-U6-pre-miR-27b into HEK293 cells and luciferase activity was measured 48 hr after transfection. Data are presented as mean±SD (N=3). The representative result from two independent experiments is shown.

Supplementary Figure S4

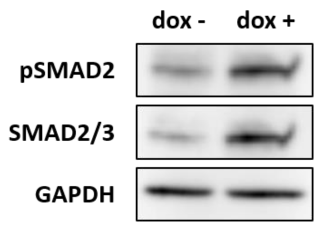

Figure S4. Phospho-SMAD2 was analyzed in hiPS-AAVS1-27b cells. hiPS-AAVS1-27b cells were differentiated as in Fig. 2A with or without dox (1 μg/ml), and Western blotting was performed at day 2. Original images are shown in Fig. S8E. The representative data from four independent experiments is shown.

Supplementary Figure S5

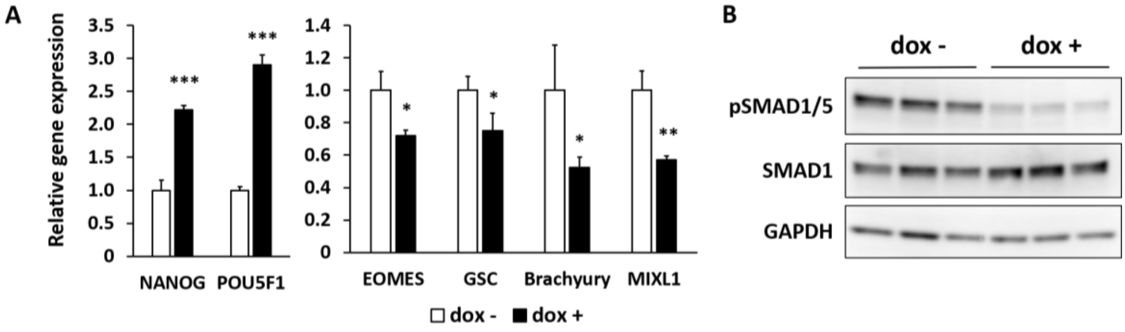

Figure S5. hiPS-AAVS-27b cell line (hiPS-AAVS-27b-10) was subjected to analysis of phosphor-SMAD1/5 protein and mRNA expression of differentiation marker genes.

(A) Expression levels of differentiation marker genes in hiPS-AAVS1-27b-10 cells were analyzed by qRT-PCR at day 4 of differentiation. hiPS-AAVS1-27b-10 cells were differentiated with or without dox (1 µg/ml). The value of dox- was taken as 1.0. Data are presented as mean±SD (N=3). Student's t-test was performed (*p<0.05, **p<0.01, ***p<0.001) The representative data from two independent experiments is shown.

(B) Expression levels of phospho-SMAD1/5 protein in hiPS-AAVS1-27b-10 cells at day 2 of differentiation were analyzed by Western blotting. Original images are shown in Fig. S8F. The representative result from two independent experiments is shown.

Supplementary Figure S6

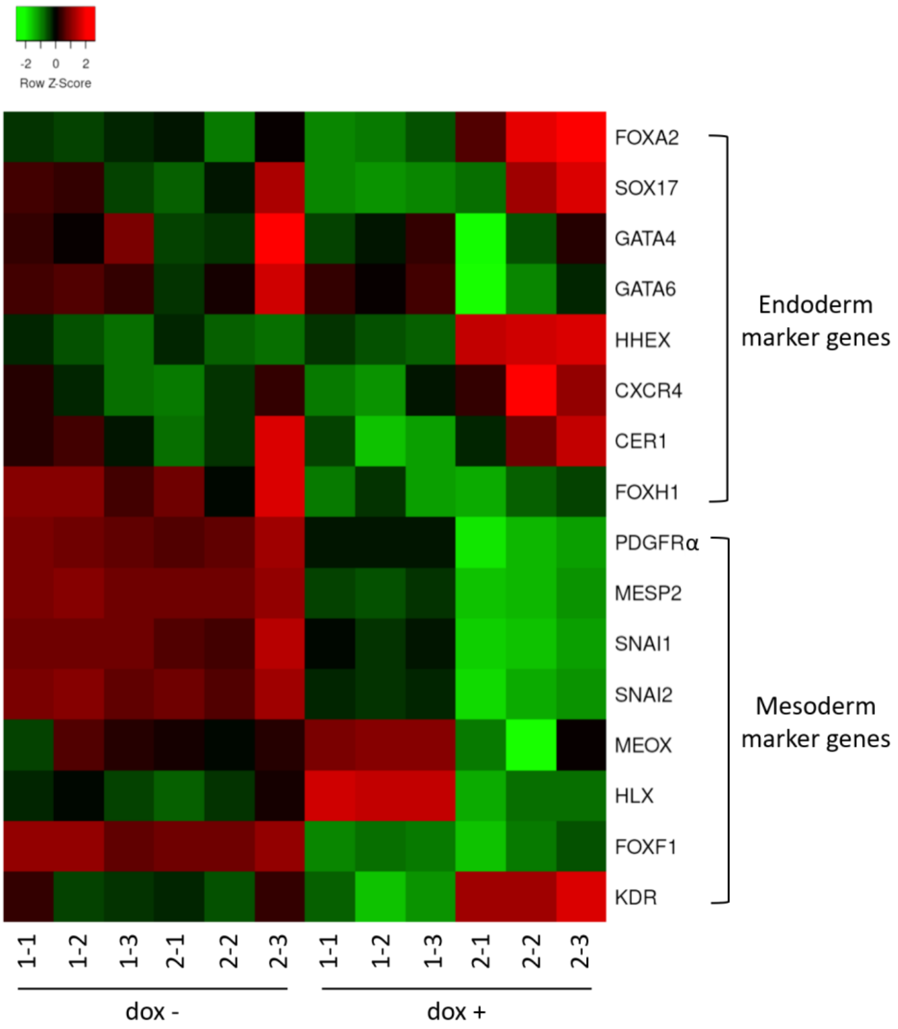

Figure S6. Expression analysis of endoderm/mesoderm marker genes at day 4. Expression levels of endoderm/mesoderm marker genes in hiPS-AAVS1-27b cells were analyzed by qRT-PCR at day 4. A heatmap represents the log2 fold-change values of these genes between dox - and dox +. The data from two independent experiments, each of which was performed in triplicate, (1-1, 1-2 and 1-3 from the experiment 1; 2-1, 2-2 and 2-3 from the experiment 2) were shown.

Supplementary Figure S7

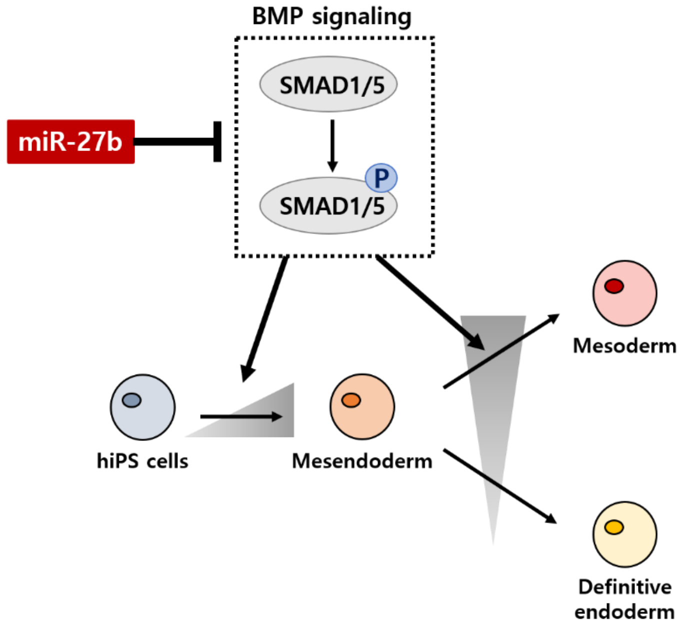

Figure S7. A schematic of the role of miR-27b in the early differentiation of hiPS cells. miR-27b antagonizes BMP signaling by lowering the phosphorylated SMAD1/5 level, which leads to decreased differentiation to mesendoderm and mesoderm. Gray triangles ~~indicated~~ the gradients of BMP signal intensity on each differentiation step.

Supplementary Figure S8

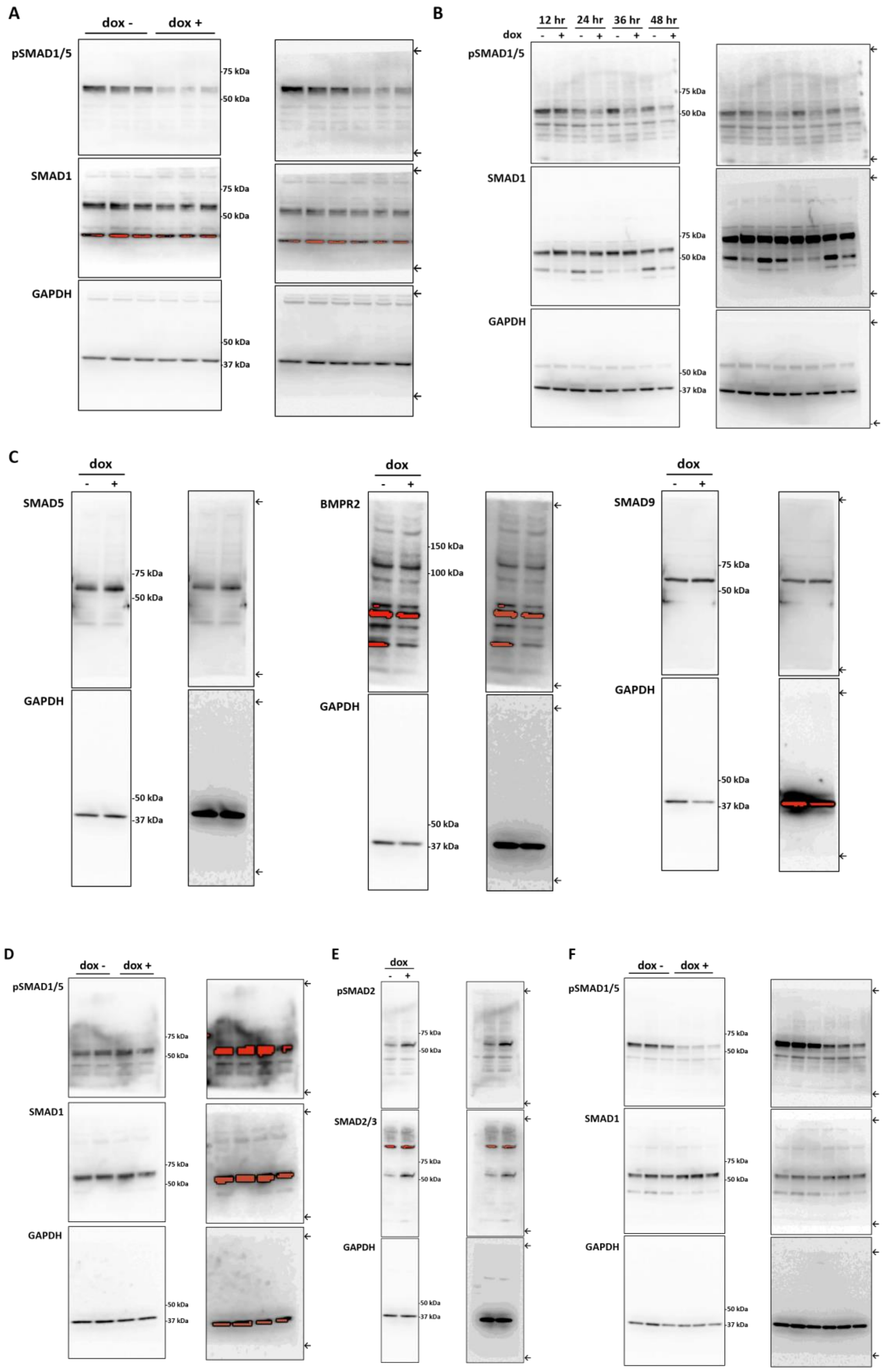

Figure S8. Full-length images of Western blot analysis. Original images (left) and longer exposed/high contrasted images (right) are shown. Membrane edges are pointed with arrows.

(A) Figure 2D

(B) Figure 3A

(C) Figure 4C

(D) Figure S2B

(E) Figure S4

(F) Figure S5B
